# Genome-wide identification and expression analysis of the WRKY gene family in *Sophora flavescens* during tissue development and salt stress

**DOI:** 10.3389/fpls.2024.1520786

**Published:** 2024-12-23

**Authors:** Jin Li, Xi Wang, Junjie Lu, Huifang Song, Haiying Lei, Tianzeng Niu, Ake Liu

**Affiliations:** ^1^ Department of Life Sciences, Changzhi University, Changzhi, China; ^2^ College of Life Science, Nanyang Normal University, Nanyang, China

**Keywords:** *Sophora flavescens*, WRKY gene family, phylogenetic analysis, tissue development, salt stress

## Abstract

*Sophora flavescens* is a traditional Chinese medicinal herb rich in various bioactive secondary metabolites, such as alkaloids and flavonoids, and exhibits remarkable resistance to abiotic stress. The WRKY transcription factor (TF) family is one of the largest plant-specific TF families and plays a crucial role in plant growth, development, and responses to abiotic stress. However, a comprehensive genome-wide analysis of the WRKY gene family in *S. flavescens* has not yet been conducted. In this study, we identified 69 *SfWRKY* genes from the *S. flavescens* genome and classified them into seven distinct subfamilies based on phylogenetic analysis. Transposed duplications and dispersed duplications were found to be the primary driving forces behind the expansion of the SfWRKY family. Additionally, several *cis*-acting elements related to the stress response and hormone signaling were discovered within the promoter regions of *SfWRKYs*. Transcriptomic analyses across five tissues (leaves, flowers, pods, roots, and stems) revealed that genes exhibiting high expression levels in specific tissues generally showed high expression across all the examined tissues. Coexpression network constructed based on metabolomic and transcriptomic analyses of root and pod development indicated that *SfWRKY29* may play a significant role in regulating the biosynthesis of secondary metabolites during tissue development. The RT-qPCR results of gene expression analysis revealed that several *SfWRKY* genes were significantly induced in response to the accumulation of secondary metabolites or salt stress. Our study systematically analyzed WRKY TFs in *S. flavescens*, which provides valuable reference data for further studies on the key roles of *SfWRKY* genes in growth development as well as their responses under salt stress conditions.

## 1 Introduction

The WRKY family is one of the largest transcription factor (TF) families, which has been reported extensively involved in regulating the biosynthesis of secondary metabolites, developmental processes and stress responses (Goyal et al., 2022; Song et al., 2023). Since the first WRKY protein structure was found in sweet potato (Ipomoea batatas *L.*) (Ishiguro and Nakamura, 1994), WRKY TFs have been extensively identified in many plants, such as *Polygonum cuspidatum, Artemisia annua, Fagopyrum tataricum* etc (Bao et al., 2018; Lv et al., 2020; Chen et al., 2021). The WRKY proteins are named for their highly conserved WRKY domain (~60 amino acids), which contains a conserved motif (WRKYGQK) located at the N-terminus, followed closely by a zinc finger motif (Eulgem et al., 2000; Cheng et al., 2019). The WRKY region consists of four lines with a β-fold composition of Zn^2+^ that coordinates with Cys/His residues to form a zinc finger structure (Yamasaki et al., 2005; Duan et al., 2007).

In terms of phylogeny, WRKY proteins are classified into three groups (I-III) based on the number of WRKY domains and the type of zinc finger structure. Group I proteins contain two WRKY domains and a C_2_H_2_ (CX_4_-_5_CX_22_-_23_HXH) zinc finger motif. In contrast, both Group II and Group III proteins possess only a single WRKY domain along with either a C_2_H_2_ or a modified C_2_HC (CX_7_CX_23_HXC) zinc finger motif (Goyal et al., 2022; Long et al., 2023). Furthermore, Group II can be subdivided into five distinct subgroups (IIa~IIe). The amino acid sequences of WRKY proteins specifically bind to the W-box *cis*-regulatory element (TTGACT/C) within target gene promoters, thereby inducing their expression. This interaction plays a crucial role in regulating plant secondary metabolite synthesis, growth and development, as well as responses to biotic and abiotic stresses (Wani et al., 2021; Zhang et al., 2018).

Increasing evidence suggested that WRKYs serve as important regulatory foundations for plant growth and development (Jiang et al., 2017; Wang et al., 2023). Under short-day conditions, WRKY12 and WRKY13 were involved in regulating the flowering time of *Arabidopsis thaliana* (Li et al., 2016; Ma et al., 2020). In rice, OsWRKY78 plays a role in stem elongation and seed development (Zhang et al., 2011). WRKY26, WRKY45, and WRKY75 participate alongside ethylene in inhibiting the growth of primary roots and lateral roots during shade-avoidance response (Rosado et al., 2022). Following treatment with As + Fe, the expression of rice *OsWRKY71* increases, promoting root system development while also participating in the regulation of gibberellin synthesis pathways (Mirza and Gupta, 2024). In both *A. thaliana* and rapeseed, *WRKY70* was primarily expressed in leaves where it plays a significant role in leaf senescence (Ülker et al., 2007; Liu et al., 2023). Furthermore, it has been demonstrated that WRKY TFs possess functions that regulate the biosynthesis of terpenoids, alkaloids, flavonoids, etc. AaWRKY1 positively regulates artemisinin biosynthesis by promoting the expression of *DBR2*, *CYP71AV1*, and *ADS* genes within *A. annua* (Han et al., 2014). VqWRKY31 activates salicylic acid defense signals, which alter the accumulation of quercetin, flavonoids, and proanthocyanidins (Yin et al., 2022). In *Coptis chinensis*, CcWRKY7, CcWRKY29 and CcWRKY32 may regulate berberine alkaloid biosynthesis (Huang et al., 2023). PeWRKY30 serves as a key factor for flavonoid biosynthesis in passion fruit (Ma et al., 2024).

Previous studies have demonstrated that WRKY TFs play a crucial role in plant defensive responses to environmental stress (Jiang et al., 2017; Goyal et al., 2022; Feng et al., 2023). The overexpression of *SlWRKY8* in tomato significantly enhances its resistance to pathogen infection and positively regulates responses to drought and salt stress (Gao et al., 2019). Furthermore, in tomato, SlWRKY57 functions as a negative regulator in the response to salt stress by directly inhibiting the transcription of salt-responsive genes (*SlRD29B* and *SlDREB2*) as well as ion homeostasis genes (*SlSOS1*) (Ma et al., 2023). Under low temperature and drought conditions, the overexpression of *PoWRKY1* in *A. thaliana* has been shown to improve seed germination activity and promote root growth in transgenic plants (Wei et al., 2021). In addition, under drought and salt stress, the overexpression of *MfWRKY40* facilitates taproot elongation, enhances osmoregulation, and improves tolerance in *A. thaliana* plants (Huang et al., 2022). In wheat, the TaWRKY plays a role in regulating the response to aluminum and manganese ion stress (Luo et al., 2024). Tomato WRKY23 can enhance the salt and osmotic stress tolerance of transgenic *Arabidopsis* by modulating the ethylene and auxin pathways (Singh et al., 2023). Overexpression of *TaWRKY17* can significantly enhance the salt tolerance of wheat (Yu et al., 2023). Therefore, WRKY TFs can regulate the growth and development and environmental adaptation from multiple dimensions.

*Sophora flavescens* (Kusen), a Chinese herbal medicine, is widely used in the treatment of inflammation, solid tumors, and analgesic effect (Lee et al., 2018; Cheng et al., 2022). The main active ingredients in *S. flavescens* are alkaloids and flavonoids (Dong et al., 2021), which are involved in treating diseases such as hepatitis, tumors, and diabetes. Comparative genomics analysis can provide us with an efficient way to identify members of certain gene family and conduct studies on their potential functions (Liu et al., 2024). Given the significant contribution of the WRKY gene family to plant stress tolerance and secondary metabolite biosynthesis, in this study, we conducted phylogenetic analysis, sequence characters, tissue-specific expressions of *WRKY* genes in *S. flavescens*. Our findings will provide insight into the mechanism of environmental adaptability and secondary metabolite biosynthesis in *S. flavescens*, and also provide reference information for molecular breeding.

## 2 Materials and method

### 2.1 *WRKY* gene identification and sequence retrieval in *S. flavescens*

The genome and protein sequences of *S. flavescens* and *Sophora moorcroftiana* were obtained from previous studies (Qu et al., 2023; Yin et al., 2023). The HMM configuration file for WRKY domain (PF03106) was downloaded from the Pfam database (El-Gebali et al., 2019). Candidate WRKY members coding in the genomes of *S. flavescens* and *S. moorcroftiana* were identified using HMMER (v3.2.1) software, with an *E*-value threshold set at 10^-2^ (Prakash et al., 2017). Only sequences containing the WRKY domain were considered as members of the WRKY family. To ensure the completeness of SfWRKY repertoire, we also examined the assembled novel transcripts obtained from the transcriptome assembly in section 2.5 for member identification. Furthermore, the *WRKY* genes in *A. thaliana* were derived from previous study (Abdullah-Zawawi et al., 2021).

### 2.2 Physicochemical properties of WRKY TFs

The *WRKY* genes of *S. flavescens* were named according to their relative positions on the chromosomes. To investigate their protein properties, we utilized the ProtParam program (ExPASy tools, http://web.expasy.org/protparam/) to estimate the molecular weights (MWs) and theoretical isoelectric points (pI).

### 2.3 Phylogenetic analysis of the WRKY family

To construct the phylogenetic tree of the WRKY members, we utilized the ClustalW program in MEGA 7 (v7.0.26) software (Kumar et al., 2016) to perform multiple sequence alignments of the WRKY domain regions from above mentioned three species. Subsequently, we employed the Neighbor-Joining (NJ) method within MEGA 7 to build the phylogenetic tree, selecting Poisson model for amino acid substitution and applying pairwise deletion for gap treatment. To ensure the accuracy, we assessed the support for each relative branch through 1000 bootstrap replicates.

### 2.4 Prediction of gene duplications and *cis*-acting regulatory elements

The DupGen_finder software (Qiao et al., 2019) was employed to conduct analysis of gene duplication patterns in *S. flavescens*. It includes whole genome duplication (WGD), tandem duplication (TD), transposed duplication (TRD), proximal duplication (PD), and dispersed duplication (DSD). The non synonymous substitution rate (*Ka*) and synonymous substitution rate (*Ks*) for TD gene pairs were calculated using KaKs_calculator3 (Zhang, 2022). The YN (Yang and Nielsen) model (Yang and Nielsen, 2000) was selected to compute the *Ka*/*Ks* ratio, which serves as an indicator of selective pressure on duplicated gene pairs. The gene density and intergenomic syntenic block analysis were conducted following the methods of Feng et al. (2024). Based on the genomic annotation information, TBtools software was utilized to obtain the upstream two kb genome sequence of *SfWRKY* genes from start codon. Subsequently, potential *cis*-regulatory elements were predicted and identified using default parameters via the PlantCARE website (Lescot, 2002).

### 2.5 Analysis of *SfWRKY* expression profiles based on transcriptome sequencing

To investigate the expression patterns of the *SfWRKY* genes in different tissues and growth stages, we collected four tissues (stem, flower, leaf, and root) of *S. flavescens* cultivated for over five years at Changzhi International Shennong Traditional Chinese Medicine Cultural Expo Park (Shangdang District, Changzhi City, Shanxi Province) on July 12, 2021. Additionally, we sampled pods at six different developmental stages between July 12 and August 6, 2021, with sampling conducted every five days. The roots of *S. flavescens* at eight distinct developmental stages, which were sown in September 2022, were collected on the 20th day of each month from April to November 2023 in Dianshang County (Lucheng District, Changzhi City, Shanxi Province). Each sample comprised three biological replicates collected in 50 mL centrifuge tubes and immediately frozen in liquid nitrogen before being stored at -80°C for further analysis. The experimental methods and analytical approaches for transcriptomics (stem, flower, leaf, and root; pod and root development) and metabolomics (pod and root development) were adapted from Zhong et al. (2024). The raw data from RNA-seq samples were archived in the NCBI database under accession number PRJNA1136989. The co-expression network of transcriptomes and metabolomes for pods and roots was constructed using R (v 4.2.2) with the WGCNA (v1.71) (Langfelder and Horvath, 2008) following the methods of Liu et al. (2024). The networks with inter-gene weight values greater than 0.3 were visualized using Cytoscape (v.3.8.2) (Otasek et al., 2019).

### 2.6 Expression analysis of *SfWRKY* genes based on RT-qPCR

The roots of *S. flavescens* cultivated in Dianshang County were collected for real-time quantitative PCR (RT-qPCR) analysis. The plants included two different cultivation years, C and S represent the *S. flavescens* sowed in 2024 and 2022, respectively (sowing occurs every April), with samples collected in July 2024. For salt stress treatment, one-year-old seedlings of *S. flavescens* were irrigated with 250 mM NaCl solution (Salt), while the normal condition (NC) irrigated with equivalent distilled water. After treatment for 14 days, leaves were collected for RT-qPCR analysis. Each sample consisted of three biological replicates were collected into 50 mL centrifuge tubes and immediately frozen in liquid nitrogen before being stored at -80°C.

Total RNA was extracted from the tissues of *S. flavescens* using the polysaccharide polyphenol total RNA extraction kit (Beijing GeneBetter, China). The integrity of the RNA was confirmed with a Nanodrop 2000 spectrophotometer (Thermo Fisher, USA). Reverse transcription was performed using the HiScript II Q RT SuperMix kit (Takara, Takara Biomedical Technology (Beijing) Co., Ltd.), followed by qPCR utilizing the SYBR qPCR Master Mix kit (Vazyme, Nanjing, China). Specific primers were designed using Primer5 software (v5.00) (**Supplementary Table S1**). *EF-1α* was utilized as the reference gene. All reactions were performed in triplicate, and the relative expression levels of genes were calculated using the 2^−△△Ct^ method. Statistical significance was assessed using Student’s *t* test.

## 3 Results

### 3.1 Sixty-nine *SfWRKY* genes identified in *S. flavescens* genome

**Figure 1:**
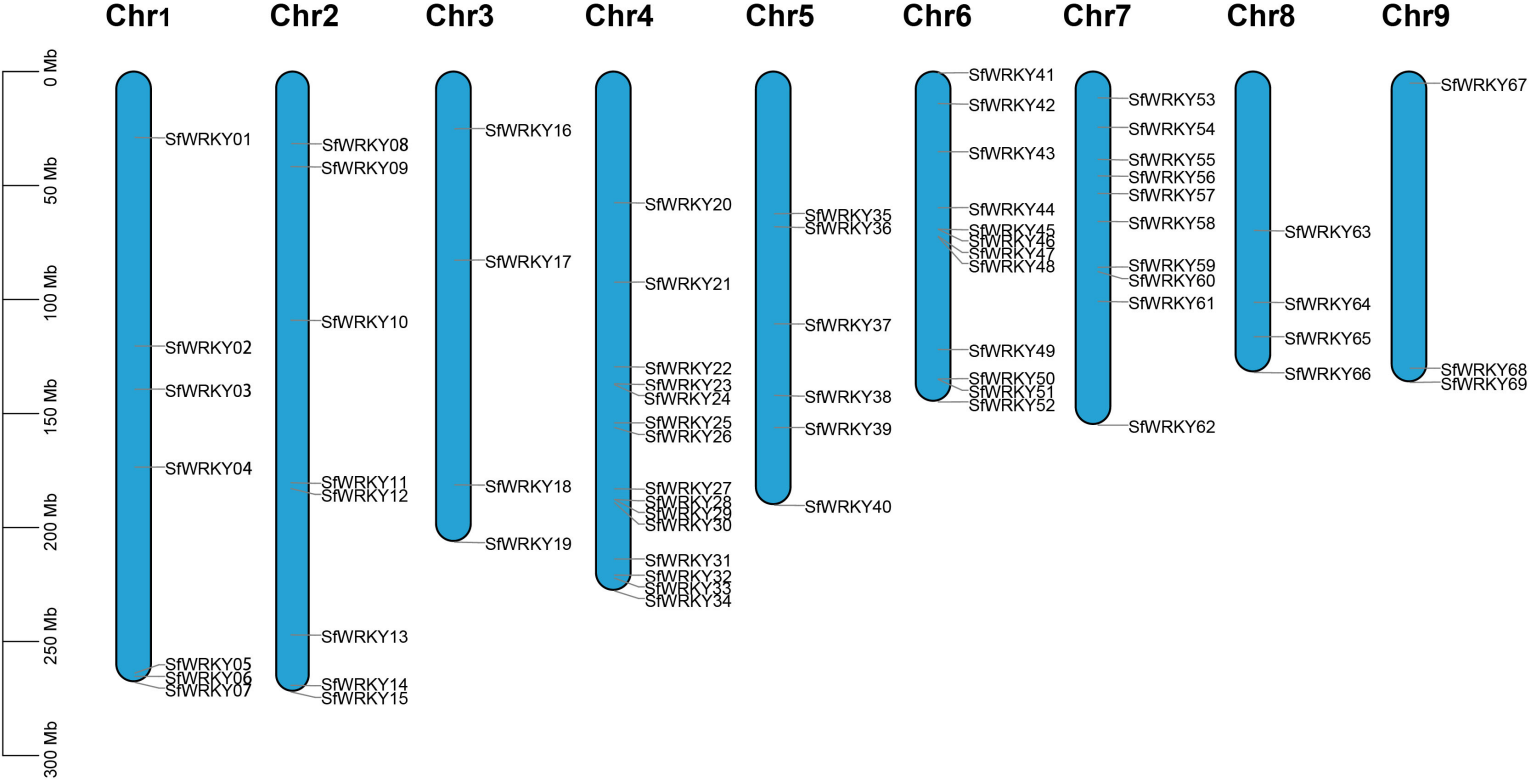
Chromosome distribution of *SfWRKY* genes. The chromosomal position of each *SfWRKY* gene was mapped according to the genome annotation file of *S. flavescens*. The chromosome number is labeled at the top of each chromosome. The scale is in mega bases (Mb) on the left.

We totally identified 69 *SfWRKY* genes based on genomic and transcriptomic data, which were named *SfWRKY01*-*SfWRKY69* according to their positions on the chromosomes (**Figure 1**; **Supplementary Table S2**). The *69 SfWRKY* genes encoded proteins of varying sizes, ranging from the largest protein (SfWRKY12) with a molecular weight (MW) of 83.5 kD and composed of 757 amino acids, to the smallest protein (SfWRKY56) with an MW of 18.8 kD and containing 167 amino acids. The theoretical isoelectric point (pI) ranges from 4.8 (SfWRKY05) to 9.78 (SfWRKY18), indicating that different SfWRKY proteins perform various functions under different microenvironments. The 69 *SfWRKY* genes are unevenly distributed across nine chromosomes of *S. flavescens* (**Figure 1**), with the majority located on chromosome 4 (Chr4, 15 genes) and Chr6 (12 genes), followed by Chr7 with 10 genes, and the least on Chr9 with only three genes.

Through correlation analysis between the *WRKY* gene numbers from the previous studies and their corresponding genome size, the former was not linearly correlated with the latter (Pearson’s correlation coefficient (*r*) = -0.0440, *p* > 0.05; **Supplementary Figure S1**). The number of *SfWRKY* genes was lower than those of *A. thaliana* (72), *S. moorcroftiana* (83), *Medicago sativa* (91), and *Arachis hypogaea* (158). However, it is relatively higher compared to other plants, such as *Panicum miliaceum* (32), *Platycodon grandiflorus* (42), and *Dendrobioum catenatum* (62). This indicates that there is no significant correlation between the size of a species’ genome and the size of the *WRKY* gene family.

### 3.2 Multiple sequence alignment, phylogenetic analysis and classification of *SfWRKY* genes

**Figure 2:**
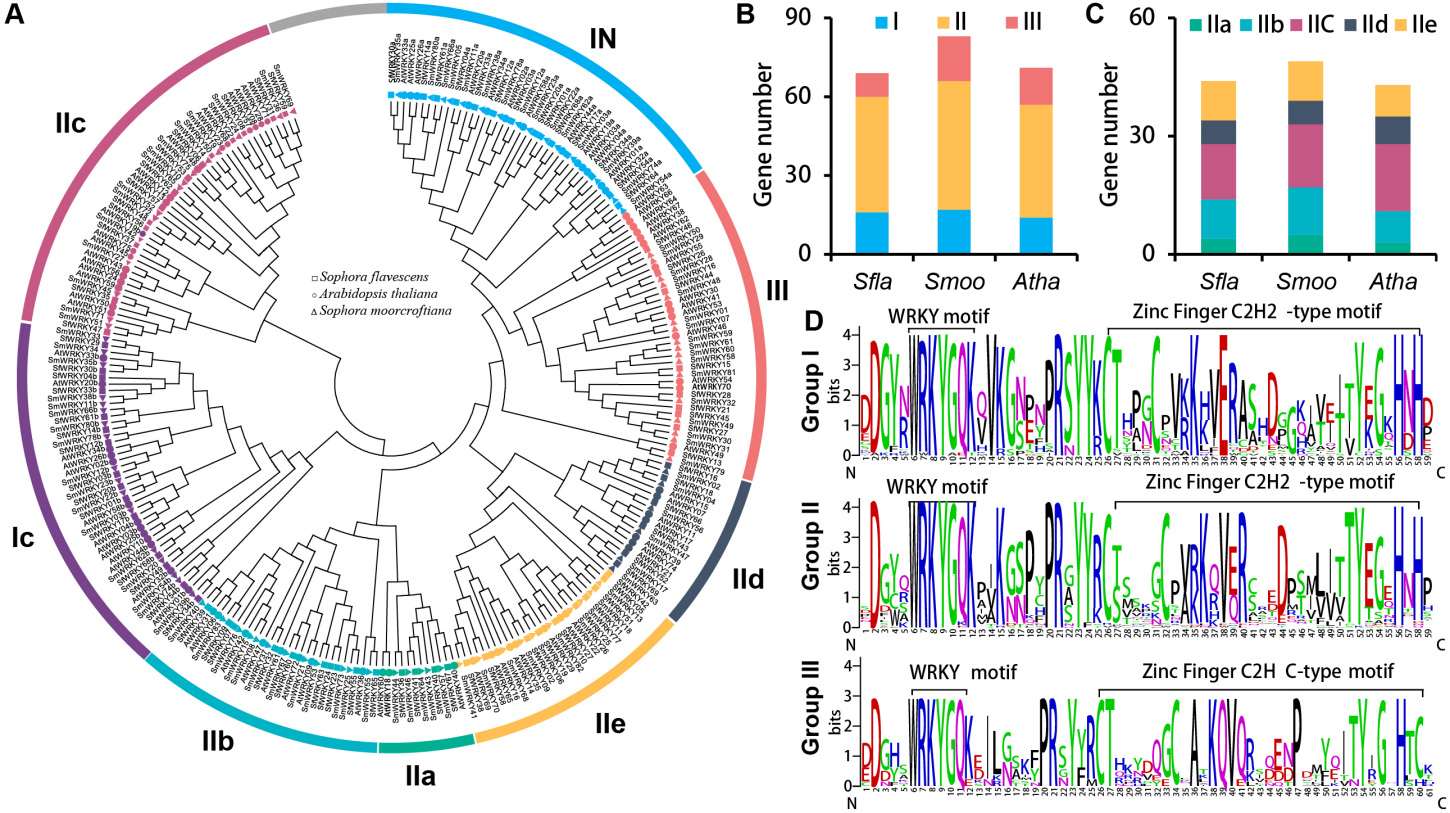
Phylogenetic analysis of *SfWRKY* genes. **(A)** Phylogenetic tree of WRKY proteins of *S. flavescens*, *S. moorcroftiana*, and *A. thaliana*. Different shapes (square, circle, and triangle) labeled at the end of each branch are used to mark the corresponding species, while different colors represent different groups. **(B)** The gene number comparison of 3 WRKY groups (I, II and III) among the *S. flavescens*, *S. moorcroftiana* and *A. thaliana*; **(C)** The member number comparison of five subfamilies in group II (IIa, IIb, IIc, IId, and IIe) among the *S. flavescens*, *S. moorcroftiana* and *A. thaliana*; **(D)** The comparative analysis of the conserved motif for the three groups of SfWRKYs based on multiple sequence alignment. The overall height of each stack represents the conservation degree of the sequence at the position. The letter at the top indicates the amino acid residue that occurs most frequently at that position.

To further understand the evolutionary diversity of the *SfWRKY* genes, we constructed an NJ tree for the WRKY family members of *S. flavescens*, *S. moorcroftiana*, and *A. thaliana* based on their WRKY domains (**Figure 2**). Accordingly, the SfWRKY members were classified into three well defined groups. The 69 *SfWRKY* genes were unevenly distributed among the three groups, with 16 members in Group I, 44 members in Group II, and nine members in Group III (**Figure 2B**). The majority of *S. flavescens* and *S. moorcroftiana* WRKY members exhibit a one-to-one clustering pattern on the evolutionary tree, whereas the *Arabidopsis* WRKY members tend to form a cohesive cluster. Members in Group I have two WRKY domains located in N-terminal and C-terminal regions. Group II has largest member number, which is further subdivided into five subfamilies (**Figure 2C**, IIa-IIe). Groups IIa and IIb tend to cluster into one branch, while groups IId and IIe tend to cluster together. In Group III, four closely located members (within 0.32 Mb) on the chromosome of *S. moorcroftiana* tend to cluster together, possibly originating from tandem duplication events. Among these groups or subfamilies, the member number in *S. flavescens* and *S. moorcroftiana* was similar (**Figures 2B, C**).

**Figure 3:**
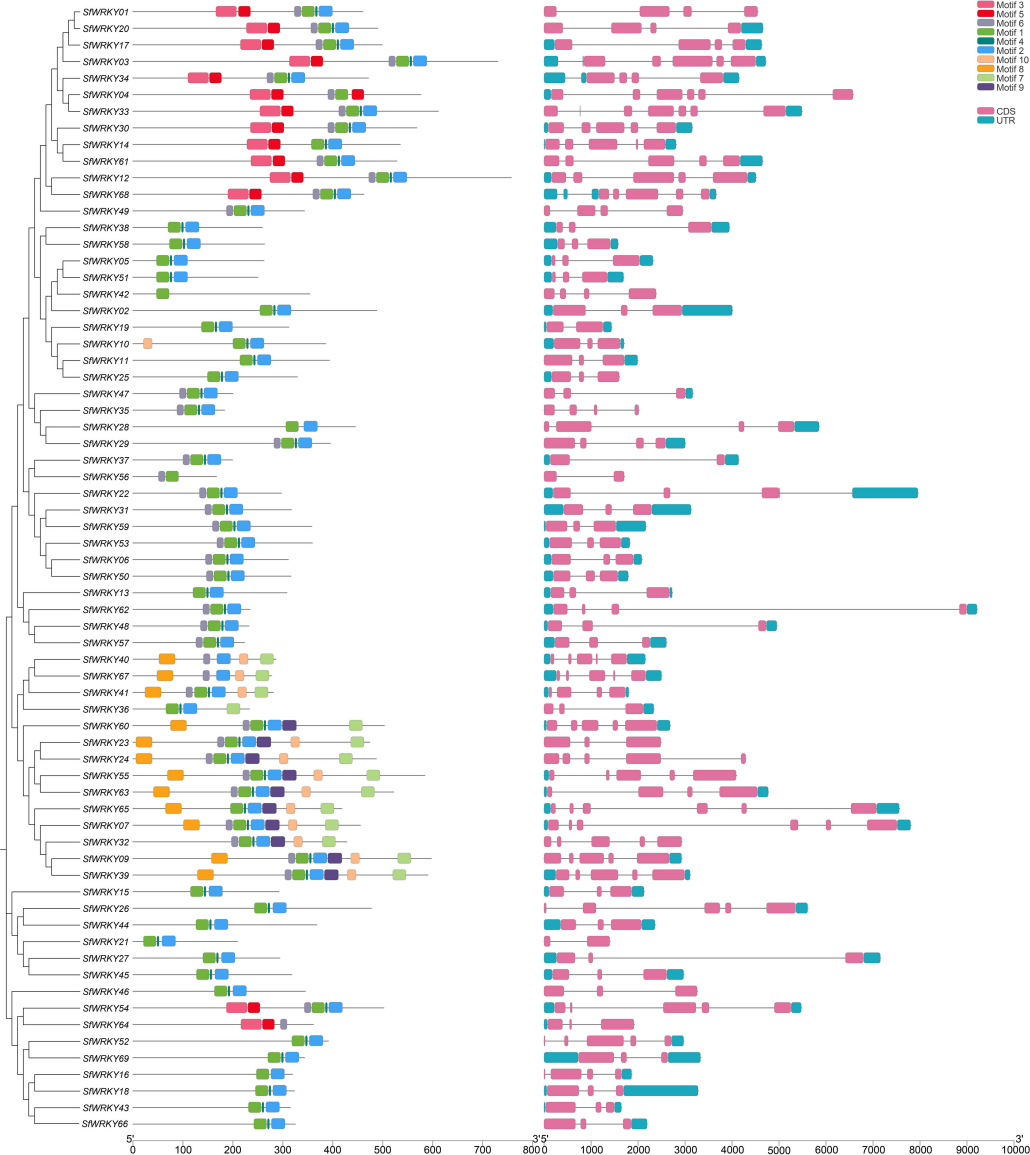
The motif distribution and gene structure of *SfWRKY* genes. The distribution of MEME motifs among the 69 WRKY members of *S. flavescens* was analyzed to unveil conserved motifs shared by related proteins. Additionally, the gene structure of these 69 *SfWRKY* genes was examined, with gray lines representing intron regions within the gene sequence. Different colors were employed to denote distinct motifs or gene regions, as depicted in the upper right corner of the figure.

Subsequently, multiple sequence alignments were conducted for the WRKY domains of the three SfWRKY groups (**Figure 2D**). The results revealed that the WRKY domains were highly conserved across all categories, containing a heptapeptide domain (WRKYGQK) and a zinc-finger domain (C2H2 or C2HC). Additionally, there were six WRKY domain variants, five of which were WRKYGEK (SfWRKY56, and SfWRKY61). The other three variants included WRKYGKK (SfWRKY35 and SfWRKY47), WKKYAQT (SfWRKY29), and WRVKGQE (SfWRKY28). The gene structure and motif distribution analyses showed members from same group or subfamily showed similar characteristics (**Figure 3**), which also provided evidence for the classification based on only phylogenetic analysis.

### 3.3 Gene duplication and collinearity of *WRKY* genes in *S. flavescens*

**Figure 4:**
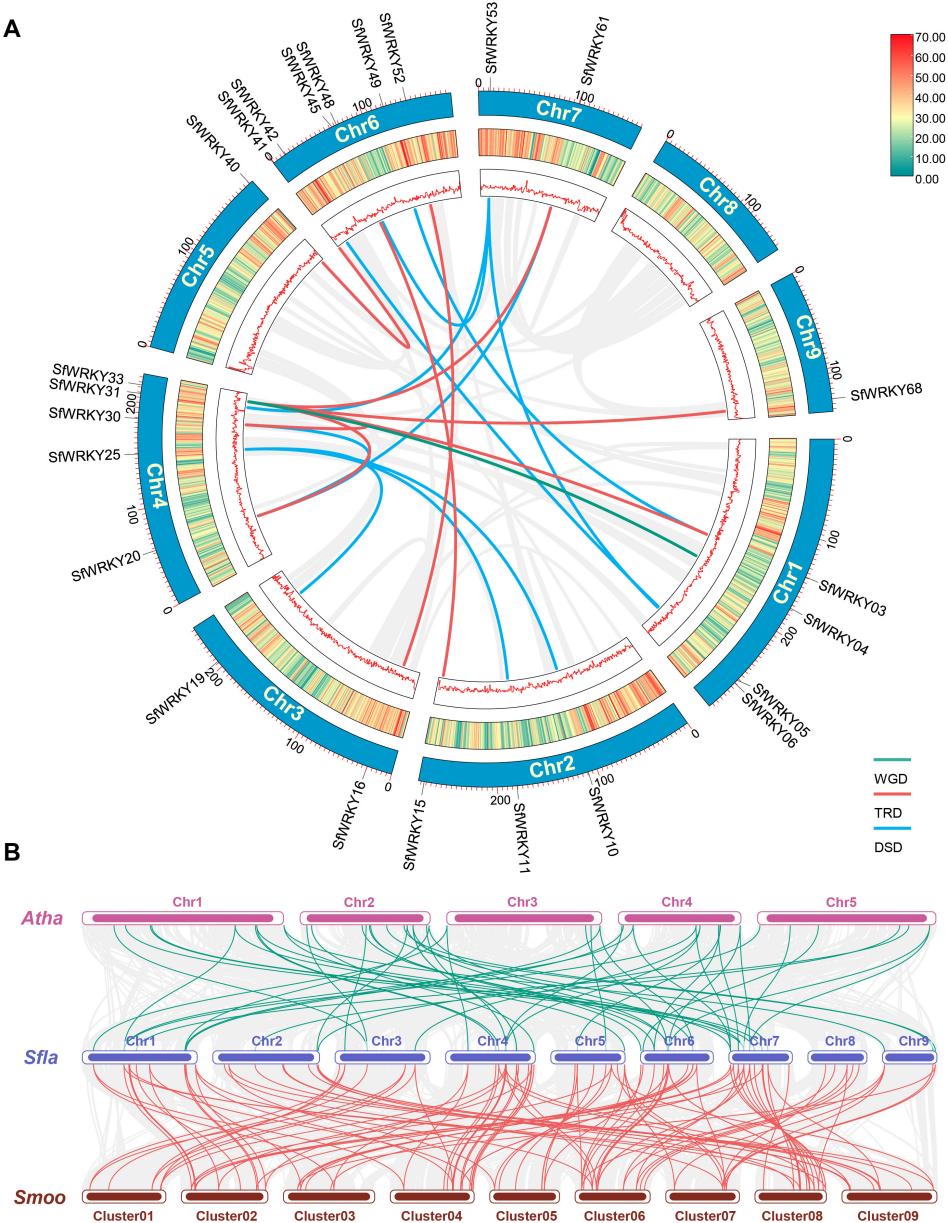
Gene duplication patterns and syntenic analyses of *WRKY* genes in *S. flavescens*, *S. moorcroftiana*, and *A. thaliana*. **(A)** The chromosome locations of duplicated *SfWRKY* genes resulted from WGD, TRD and DSD events on the Circos diagram. The different colors (red to yellow to green) in the middle track indicated high to low gene density (the number of genes per bin determined by sliding window analysis). **(B)** The intergenomic syntenic blocks identified among *A. thaliana* (Atha), *S. flavescens* (Sfla), and *S. moorcroftiana* (Smoo). The collinear blocks are represented by gray lines, while the *WRKY* gene collinearity is highlighted by green and red lines.

The DupGen_finder was used for analysis of *SfWRKY* gene duplication pattern, which included one pair of genes derived from WGD (such as *SfWRKY04* and *SfWRKY33*), 8 pairs from TRD (such as *SfWRKY20* and *SfWRKY33*), and 10 pairs of DSD (such as *SfWRKY61* and *SfWRKY20*) events (**Figure 4A**). Among these TRD and DSD gene pairs, it is generally presenting one gene corresponding to multiple duplicate genes rather than one corresponding to one, which may be related to multiple rounds of duplication occurred in *S. flavescens* genome. For example, the TRD genes corresponding to *SfWRKY33* are *SfWRKY03*, *SfWRKY20*, *SfWRKY30*, *SfWRKY61*, and *SfWRKY68*, and the DSD genes corresponding to *SfWRKY53* are *SfWRKY06*, *SfWRKY31*, and *SfWRKY48*. We further calculated the *Ka*, *Ks*, and *Ka*/*Ks* values for the duplicated *SfWRKY* gene pairs, and the results showed that the *Ka*/*Ks* values ranged from 0.09 to 0.39 (< 1), indicating that these genes underwent strong purifying selection during evolution (**Table 1**). This implies that gene duplications may contribute to the diversification and expansion of the SfWRKY gene family, while these duplicated genes are subject to strong functional constraints.

**Table 1:** Synonymous and nonsynonymous substitution rates for the duplicated gene pairs among *S. flavescens WRKY* genes.

Duplicate gene 1	Duplicate gene 2	Duplication pattern	*Ka*	*Ks*	*Ka*/*Ks*
*SfWRKY04*	*SfWRKY33*	WGD	0.127513	0.416783	0.305945
*SfWRKY11*	*SfWRKY25*	DSD	0.186373	0.531341	0.350759
*SfWRKY41*	*SfWRKY40*	TRD	0.330525	1.78668	0.184994
*SfWRKY19*	*SfWRKY25*	DSD	0.563535	2.83681	0.198651
*SfWRKY06*	*SfWRKY53*	DSD	0.452041	2.89165	0.156326
*SfWRKY48*	*SfWRKY53*	DSD	0.625776	3.7787	0.165606
*SfWRKY16*	*SfWRKY52*	TRD	0.526062	3.89375	0.135104
*SfWRKY15*	*SfWRKY45*	TRD	0.630472	3.92101	0.160793
*SfWRKY31*	*SfWRKY53*	DSD	0.538184	3.99948	0.134563
*SfWRKY10*	*SfWRKY25*	DSD	0.523031	4.0695	0.128525
*SfWRKY68*	*SfWRKY33*	TRD	0.625876	4.20783	0.148741
*SfWRKY20*	*SfWRKY33*	TRD	0.497031	4.21263	0.117986
*SfWRKY61*	*SfWRKY33*	TRD	0.576182	4.22011	0.136532
*SfWRKY03*	*SfWRKY33*	TRD	0.513669	4.30337	0.119364
*SfWRKY30*	*SfWRKY33*	TRD	0.561847	4.31147	0.130314

We also conducted a collinearity analysis among the three species, *S. flavescens*, *S. moorcroftiana*, and *A. thaliana*. The results showed that 16 *SfWRKY* genes from *S. flavescens* are present in collinear blocks with 11 counterparts from *A. thaliana*, and 24 *SfWRKY* genes from *S. flavescens* are present in collinear blocks with 12 counterparts from *S. moorcroftiana* (**Figure 4B**). It was observed that collinear genes tended to cluster into the same groups in phylogenetic tree (**Figure 2**), implying that they have a common evolutionary origin.

### 3.4 *Cis*-regulatory element distribution of *SfWRKY* genes

**Figure 5:**
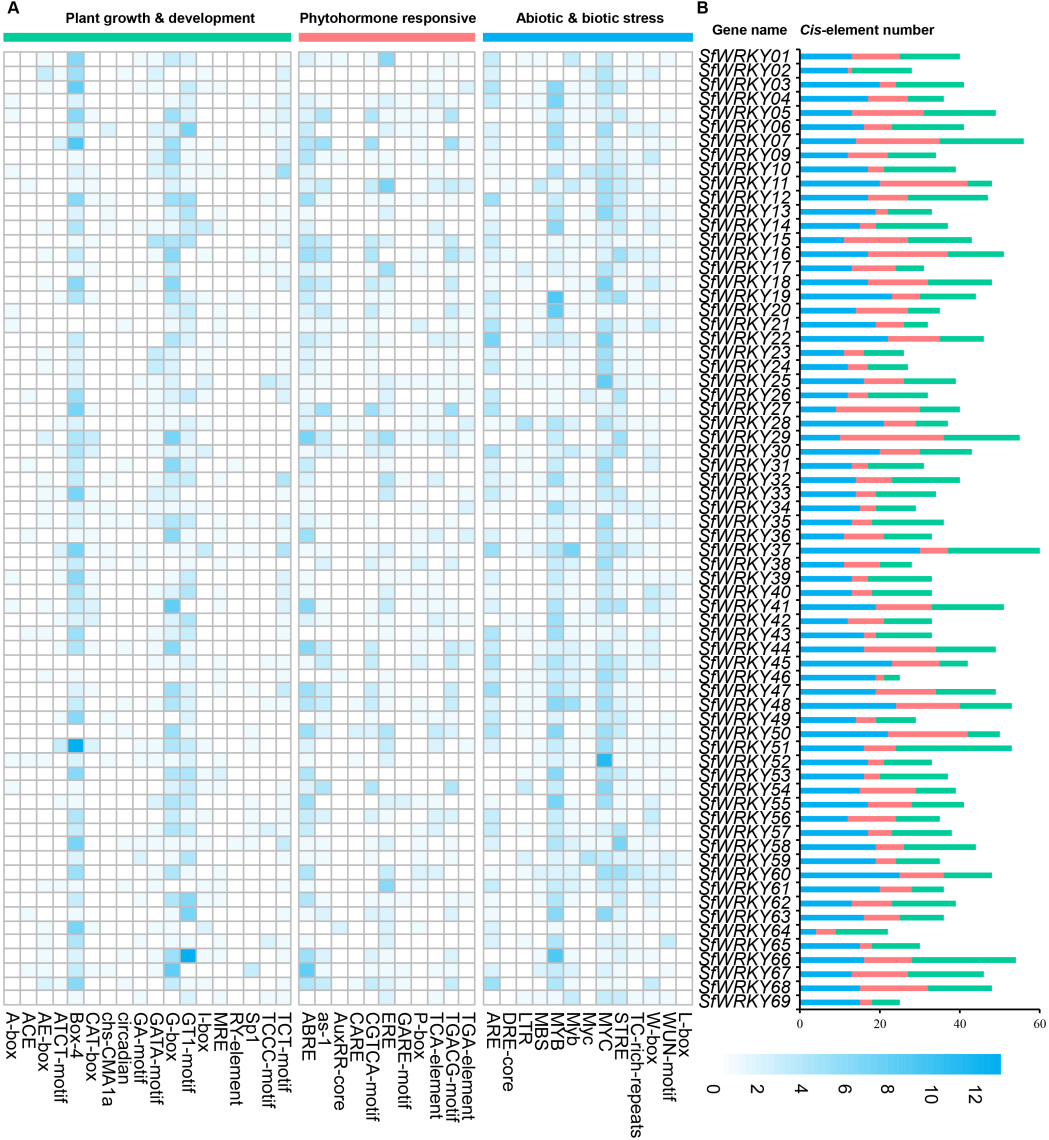
Analysis of the *cis*-elements located in the promoter regions of *SfWRKY* genes. **(A)** The different intensity colors indicate the numbers of different *cis*-elements in the promoter regions of *SfWRKY* genes (upstream 2kb region). **(B)** The different colored histograms represent the sum of the *cis*-acting elements in each category.

To understand the potential roles of the SfWRKY family members in plant growth and development, response to plant hormones, and environmental stresses, we analyzed the distribution of *cis*-elements in the upstream promoter regions of the *SfWRKY* genes (**Figure 5**). Totally, 18 plant growth and development related *cis*-elements were identified. The most abundant elements were the Box-4 (ATTAAT) and the G-box (TACGTG), which were involved in light responsiveness. They accounted for 30% and 20% of the total elements identified in this category. *SfWRKY51* contains the highest number of such elements (13). Other elements were also observed, such as circadian control and tissue-specific motifs like the GT1-motif (photosynthetic reaction regulation), TCT-motif (light-responsive elements), and GATA-motif (plant development). Regarding the phytohormone responsive elements, the ABRE element involved in ABA responsiveness, the TCA-element involved in salicylic acid responsiveness, the TGACG-motif involved in MeJA-responsiveness and the ERE element involved in ethylene signal regulation were identified abundant in upstream regions of the *SfWRKY* genes. These four pattern elements represent more than 45% of the total hormone-responsive elements. In *SfWRKY67*, the number of ABRE elements is the highest (8), while in *SfWRKY11*, the number of ERE elements is the highest (7). In addition, other elements were also found, like the as-1 element involved in SA and oxidative stress responsiveness, and the CGTCA-motif involved in MeJA-responsiveness in this category.

In the abiotic and biotic stress category, different elements associated with stress responses, such as oxidation, defense, drought, wounding, heat, and low temperature, were observed. After our analysis in *S. flavescens*, the largest part of the elements belonging to the abiotic and biotic stress category corresponds to two general stress-responsive motifs, namely the MYB (CCAAT box) and MYC (CACATG box) binding sites, representing 30% and 20% of the total identified *cis*-elements, respectively. In addition, other stress-specific *cis*-elements were identified. Several of them were responsive to wounding and pathogens—including the WRKY-box (W-box), the TC-rich repeats, and the wound responsive motif (WUN-motif). Temperature-related elements, such as the stress responsive element (STRE) and the low-temperature responsive (LTR) motifs; drought related elements, such as the MYB BINDING SITE (MBS) and the Dehydration -responsive element (DRE)-core; and anaerobic conditions like the Anaerobic response element (ARE) motif (**Figure 5**). Among them, *SfWRKY37* contains the highest number of such elements, followed by *SfWRKY60*.

### 3.5 Expression profiles of *SfWRKY* genes among different tissues

**Figure 6:**
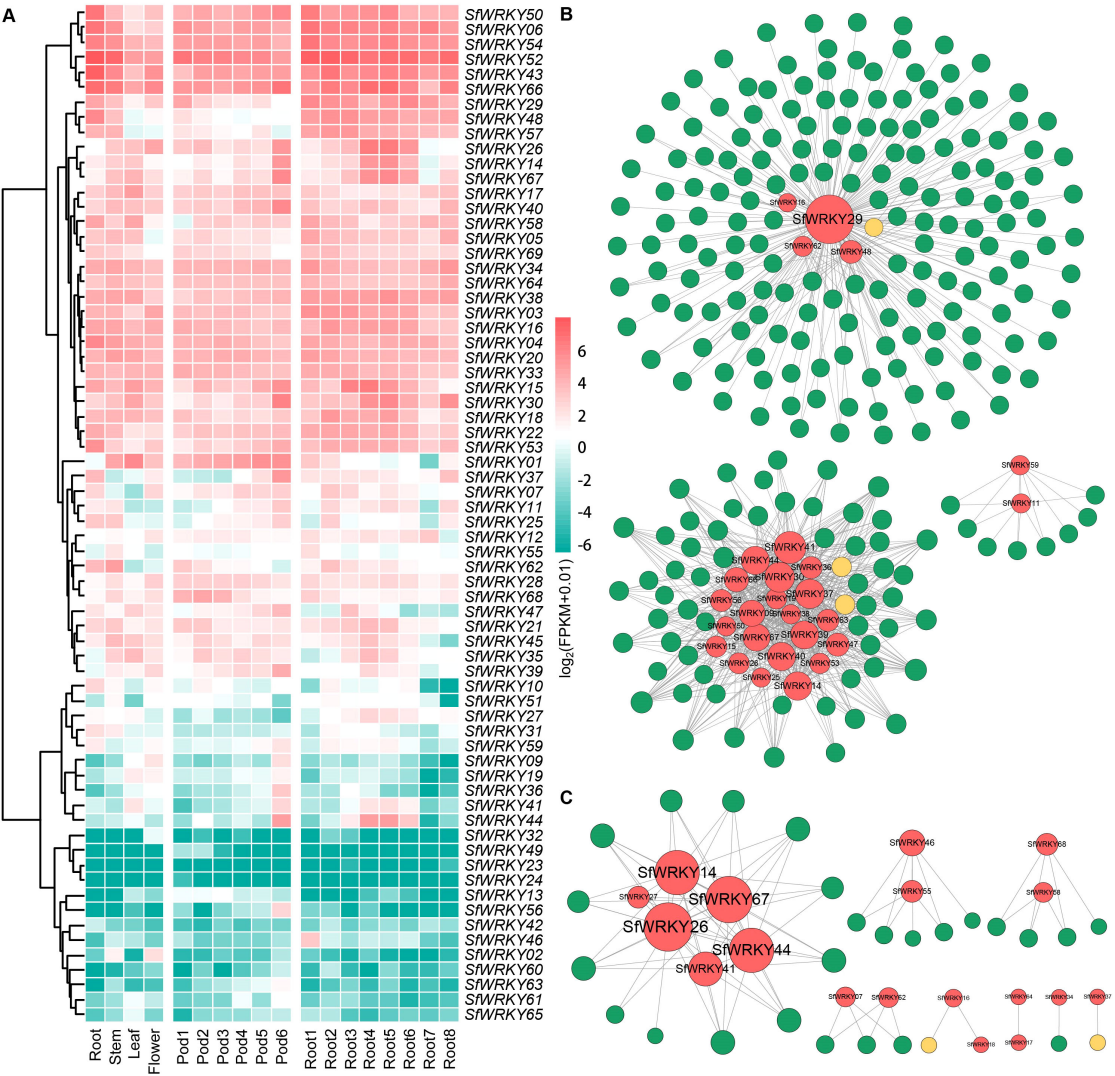
Expression patterns of *SfWRKY* genes among different tissues. **(A)** Expression profiles of the *SfWRKY* genes among different tissues or developmental stages. **(B)** Coexpression network of *SfWRKY* genes reconstructed based on transcriptome and metabolome during the pod development. **(C)** Coexpression network of *SfWRKY* genes reconstructed based on transcriptome and metabolome during the root development.

To investigate the expression patterns of SfWRKY family members among different tissues, we analyzed their expression levels in four different tissues, including leaves, flowers, roots, and stems, as well as different development stages of pods and roots. The results showed significant variations in the expression patterns of different *SfWRKY* genes across various tissues (**Figure 6**). Notably, *SfWRKY52* and *SfWRKY66* exhibited consistently high expression levels throughout all examined tissues and developmental stages. We can classify the expression patterns of *SfWRKY* genes among different tissues mainly into three categories based on the tissue expression profiles: the first category includes genes with high expression levels, such as *SfWRKY52*, *SfWRKY66*, *SfWRKY43*, and *SfWRKY52*, which are highly expressed among the root, stem, leaf, and flower tissues, and across all the six developmental stages of the pod tissue. The second category includes low-expressed genes among many tissues, such as *SfWRKY28*, *SfWRKY68*, and *SfWRKY47*, among all the four tissues; *SfWRKY12* and *SfWRKY28* across the pod tissues; and *SfWRKY12*, *SfWRKY28*, and *SfWRKY21* across the root tissues. The third category includes genes that do not express, such as *SfWRKY61*, *SfWRKY63*, and *SfWRKY62*, which are not expressed in any of the five tissues. It is noteworthy that the genes exhibiting high expression levels in one tissue or nearly ubiquitous high expression across all tissues, implying their predominant involvement in plant growth and development.

The gene coexpression networks were constructed based on transcriptome and metabolome data obtained from developmental stages of *S. flavescens* pods and roots. The results revealed that *SfWRKY29* and *SfWRKY41* were identified as hub-genes among the pod network (**Figure 6B**), indicating their pivotal roles in regulating the biosynthesis of secondary metabolites during pod development. IFR (2’-hydroxyisoflavanone reductase) and IFS (2-hydroxyisoflavanone synthase) emerged as key enzymes within the highly interconnected metabolic pathways. We further analyzed the coexpression network constructed based on the transcriptomics and metabolomics of *S. flavescens* root development, revealing *SfWRKY29* as a central hub with the highest connectivity to other genes (**Figure 6C**), indicating its crucial involvement in regulating biosynthesis of secondary metabolites during root development. Notably, only I2’H (isoflavone 2’-hydroxylase) and I3’H (isoflavone 3’-hydroxylase) were identified as pivotal enzymes operating within this metabolic pathway.

### 3.6 Expression analysis of *SfWRKY* genes in *S. flavescens* roots with different cultivated years based on RT-qPCR

**Figure 7:**
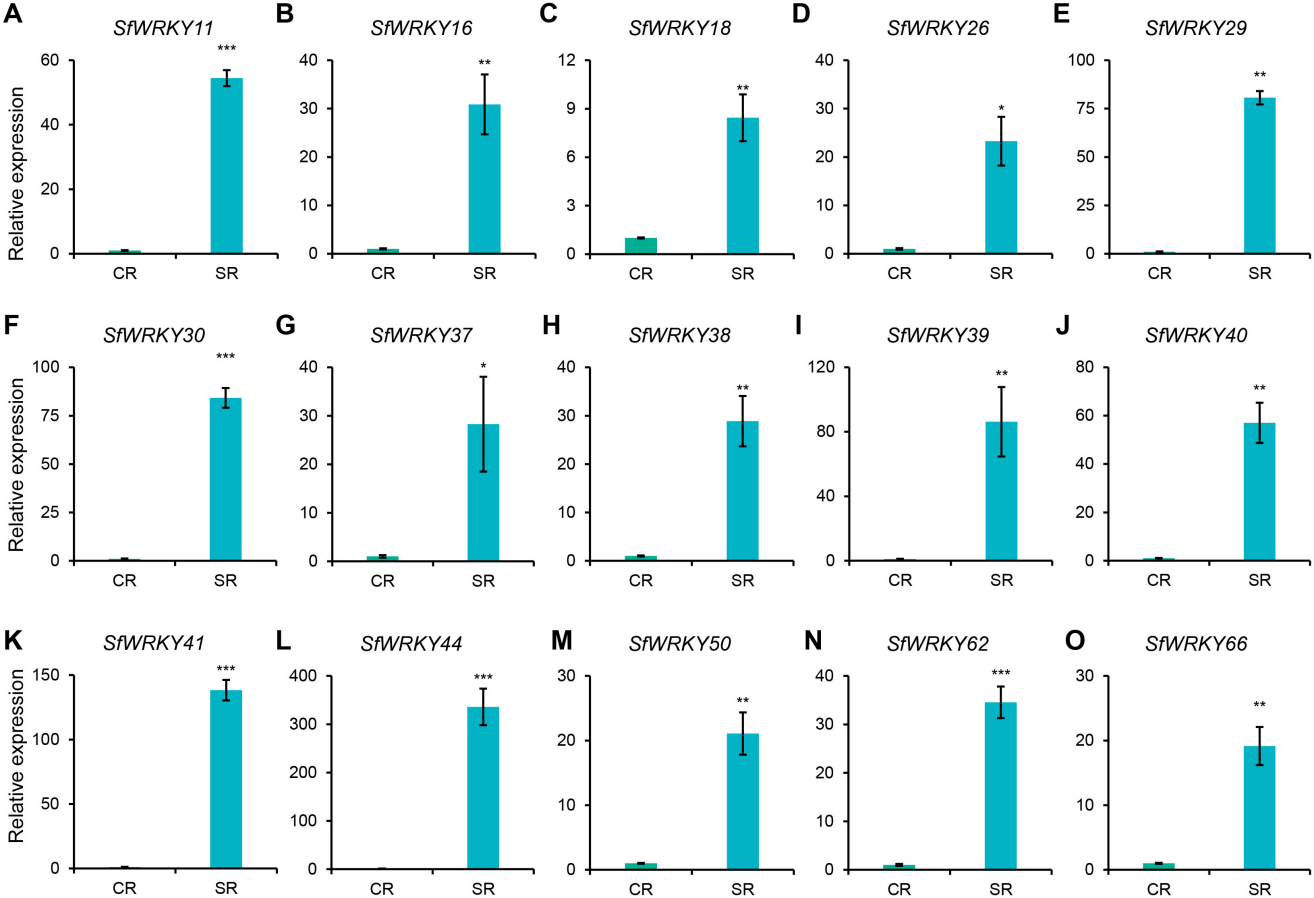
Expression analysis of *SfWRKY* genes in S. flavescens roots with different cultivated years by RT-qPCR. Panels **(A–O)** represent the relative expression of *SfWRKY11*, *SfWRKY16*, *SfWRKY18*, *SfWRKY26*, *SfWRKY29*, *SfWRKY30*, *SfWRKY37*, *SfWRKY38*, *SfWRKY39*, *SfWRKY40*, *SfWRKY41*, *SfWRKY44*, *SfWRKY50*, *SfWRKY62*, and *SfWRKY66* genes, respectively. Each sample included three biological replicates. The symbols *, **, and *** above the error bar indicate a statistically significant difference between CR (roots from S. flavescens sowed in current years) and SR (roots from S. flavescens sowed two years ago) samples at p < 0.05, p < 0.01, and p < 0.001 significance level.

As we know, the roots of *S. flavescens* can accumulate secondary metabolites such as flavonoids and alkaloids as they grow, and the accumulation increases with the growing years (Lei et al., 2021). Excessive accumulation often leads to autotoxicity, which inhibits plant growth or causes continuous monocropping obstacle. Given the outstanding performance of *WRKY* genes in resistance to adverse conditions, we used RT-qPCR technology to analyze the expression patterns of WRKY family members in the roots of *S. flavescens* with cultivated years. As shown in **Figure 7**, among the 15 selected *SfWRKY* genes, all showed significantly higher expression in the SR (roots from *S. flavescens* sowed two years ago) than in the CR (roots from *S. flavescens* sowed in current years), with the greatest expression difference being observed in *SfWRKY44* (upregulated by more than 335 times), followed by *SfWRKY41* and *SfWRKY39*. Other *SfWRKY* genes also showed varying degrees of significant upregulation. This result implies that *WRKY* genes in *S. flavescens* play an important role in the response to the accumulation of secondary metabolites.

### 3.7 Expression analysis of *WRKY* genes in leaves of *S. flavescens* under salt stress based on RT-qPCR

**Figure 8:**
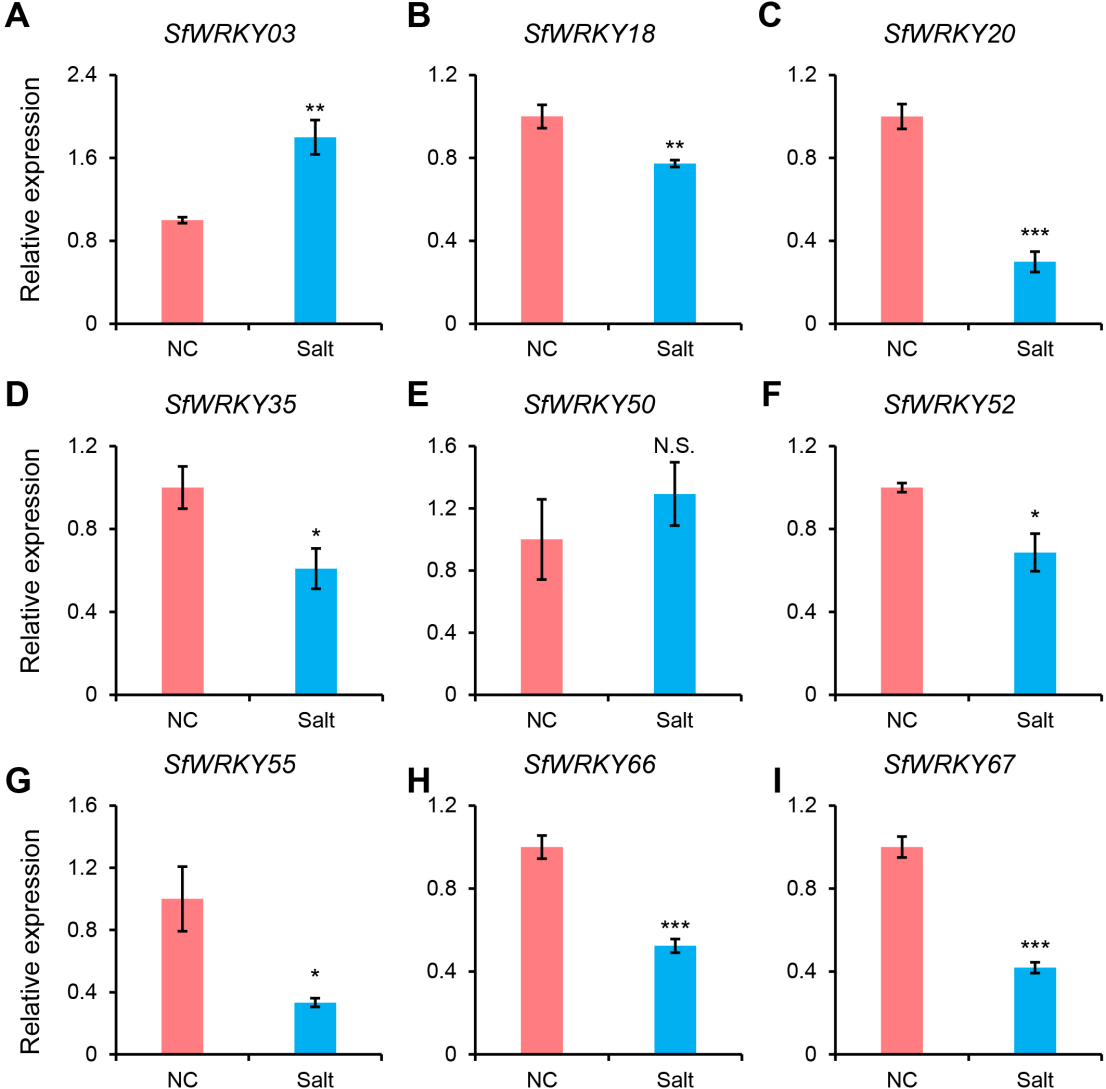
Expression analysis of *SfWRKY* genes in the leaves of S. flavescens under salt stress by RT-qPCR. Panels **(A–I)** represent the relative expression of *SfWRKY03*, *SfWRKY18*, *SfWRKY20*, *SfWRKY35*, *SfWRKY50*, *SfWRKY52*, *SfWRKY55*, *SfWRKY66*, and *SfWRKY67* genes, respectively. Each sample contain three biological replicates. The symbols *, **, and *** above the error bar indicate a statistically significant difference between the NC (normal condition) and Salt (250 mM NaCl) treatments at p < 0.05, p < 0.01, and p < 0.001 significance level (Student’s t test). N.S., not significant.

To investigate the expression patterns of WRKY family members in the leaf tissues of *S. flavescens* under salt stress, we used RT-qPCR to analyze the expression patterns of 9 *SfWRKY* genes (**Figure 8**). The results showed that seven *SfWRKY* genes (*SfWRKY18*, *SfWRKY20*, *SfWRKY35*, *SfWRKY52*, *SfWRKY55*, *SfWRKY66*, and *SfWRKY67*) were downregulated under salt stress, while two genes (*SfWRKY03* and *SfWRKY50*) were upregulated. The downregulation of *SfWRKY55* was the most significant, while the *SfWRKY03* was upregulated by 1.8 times when exposed to salt stress. Their significant expression differences under salt stress may suggest that they are involved in the response of *S. flavescens* to salt stress.

## 4 Discussion

*S. flavescens* is an herbaceous plant whose roots are often used in traditional Chinese medicine. It contains various active ingredients, including alkaloids, flavonoids, and others, which have shown significant effects in treating inflammation, fever, cancer, and skin diseases (He et al., 2015; Gao et al., 2024). *S. flavescens* also has a wide suitable growth range, including in barren gullies, shrublands, or fields. The plants exhibited robust stress resistance, demonstrating practical cold tolerance, heat resistance, and saline-alkali tolerance. It has been reported that the members of the WRKY family participate in various stress regulation networks in plants (Jiang et al., 2017; Goyal et al., 2022). Recently, studies were mainly focused on the analysis of effective components in *S. flavescens* and their related therapeutic mechanisms (Kong et al., 2024; Lin et al., 2024), but the regulatory roles of WRKY TFs in the growth and development of *S. flavescens*, especially in the regulation of stress conditions, are not clear. In this study, we conducted a comprehensive analysis of the 69 *S. flavescens* WRKY members based on the recently published genome of *S. flavescens* (Qu et al., 2023), suggesting the evolutionary diversity and complexity of the *SfWRKY* gene family. It has a significantly lower count than the 74 *WRKYs* in *Arabidopsis* and the 109 *WRKYs* in *Oryza sativa* (**Supplementary Table S3**) (Abdullah-Zawawi et al., 2021) but higher than those in *Nelumbo nucifera* (65), *Ananas comosus* (54), and *Glycyrrhiza uralensis* (52) (Xie et al., 2018; Li et al., 2019; Xiao et al., 2024).

Our evolutionary analysis classified the *SfWRKY* genes into three main groups, with the second group having the highest number of members, further divided into five subgroups, consistent with classifications in other plants (Eulgem et al., 2000; Rushton et al., 2010; Liu et al., 2020). Following the well-defined classification, the SfWRKY gene family was subdivided into three groups (**Figure 2A**). Notably, Group II contains the highest number of *SfWRKY* genes, which may be attributed to gene duplication. In Group II, a total of 11 gene duplications were identified (**Table 1**). It also revealed extensive collinearity between *S. flavescens*, *A. thaliana*, and *S. moorcroftiana*, suggesting a common ancestor before the divergence of these lineages. The variation in collinearity between *S. flavescens* and *S. moorcroftiana*, compared to *A. thaliana*, is consistent with their evolutionary relationships. The presence of conserved motifs in *SfWRKY* genes supports their functional conservation across different plants. Gene and genome duplications have long been considered as a fundamental source of evolutionary innovation, offering an expanded molecular reservoir for the adaptive evolution of key pathways, plant development, and ecological transitions (Panchy et al., 2016). Previous studies suggest that the expansion of the WRKY gene family was mainly due to tandem and segmental duplication events (Chen et al., 2019). Gene duplication analysis suggests that TRD and DSD have greatly contributed to the expansion of SfWRKY gene family (**Table 1**). Selection pressure analysis showed that *SfWRKYs* have undergone purifying selection, offering an explanation for the observed differences. The *Ka*/*Ks* ratio calculations for all inferred duplicated genes were less than one. Therefore, these gene pairs may have experienced negative selection after duplication, with limited functional divergence, indicating stable changes in amino acid sequences and subfuncitionalization during the evolution of *S. flavescens* (Cusack and Wolfe, 2007).

WRKY TFs are crucial in the regulation of gene expression, as they specifically bind to the W-box motif located within the promoter regions of target genes (Jiang et al., 2017). Similar to other plant species, the majority of SfWRKY proteins possess a WRKYGQK domain. Nevertheless, multiple variants of the *SfWRKY* gene have been identified (**Figure 5**); for instance, the WRKYGKK variant in soybean failed to effectively bind to the W-box (Zhou et al., 2008). The similarities in characteristics between SfWRKY35 and SfWRKY47 in *S. flavescens* require further investigation.

The *cis*-acting elements within gene promoters are crucial for understanding gene regulation, as they interact with transcription factors (Hernandez-Garcia and Finer, 2014). The promoters of *SfWRKY* genes contain various *cis*-acting elements that are closely associated with stress responses, plant hormone signaling, and plant growth and development. This indicates their significant roles in the response to both biotic and abiotic stresses. Previous study indicates that the expression of *WRKY* genes in specific tissues significantly influences plant growth and development (Wang et al., 2023). In this study, we observed significant differences in the expression levels of various *SfWRKY* genes across the leaves, flowers, pods, roots, and stems of *S. flavescens*. Among these, *SfWRKY52* and *SfWRKY66* exhibited consistently high expression levels across all the tissues and developmental stages (**Figure 6A**), suggesting their crucial roles involved in plant growth and development. Furthermore, through a coexpression network analysis of transcriptomic and metabolomic data from the pods and roots of *S. flavescens*, we identified *SfWRKY29* as a core gene. This finding implies its key regulatory role in biosynthesis during pod and root development.

The *WRKY* gene family plays a crucial role in regulating plant responses to various abiotic and biotic stresses (Jiang et al., 2017). To investigate the response of *SfWRKY* genes under salt stress, we analyzed their expression levels. The results indicated that eight *SfWRKY* genes exhibited differential expression under salt stress conditions. Overexpression of the *GmWRKY34* gene in *A. thaliana* significantly enhanced the plants’ salt tolerance (Zhou et al., 2015), while *CdWRKY2* was found to negatively regulate lateral root growth under salt stress (Shao et al., 2023). Therefore, we hypothesize that these eight *SfWRKY* genes may be involved in the regulation of leaf responses to salt stress in *S. flavescens*. Of course, more studies are required to provide functional validation of *SfWRKY* genes through molecular biology techniques.

## 5 Conclusion

Our study conducted a comprehensive analysis of the SfWRKY gene family in *S. flavescens* through bioinformatic methods. A total of 69 *SfWRKY* genes were identified and classified into seven subfamilies (I, IIa, IIb, IIc, IId, IIe, and III), and characterize the physicochemical properties, chromosomal locations, phylogenetic relationships, synteny features, gene structures and *cis*-regulatory elements were characterized. Coexpression analysis of the transcriptomes and metabolomes from different tissues or different stages, it was found that *SfWRKY29* exhibited the highest connectivity with other genes, indicating that it plays a crucial role in regulating the biosynthesis of secondary metabolites. The RT-qPCR results of gene expression analysis revealed that some *SfWRKY* genes of *S. flavescens* were significantly induced in response to the accumulation of secondary metabolites or salt stress. Our study would lay a foundation for understanding the roles of *WRKY* genes in the growth and development of *S. flavescens* as well as their molecular mechanisms under abiotic stress.

## Data Availability

The datasets presented in this study can be found in online repositories. The names of the repository/repositories and accession number(s) can be found in the article/**Supplementary Material**.

## Supplementary material

Supplementary Figure 1 Relationship between WRKY numbers in reported species and their corresponding genome sizes.

Supplementary Table 1 Primer sequences used for RT-qPCR.

Supplementary Table 2 Detailed information of the *S. flavescens WRKY* gene family.
