# Supplementary figures and images for: Genome-wide identification and expression analysis of the WRKY gene family in Sophora flavescens during tissue development and salt stress
Source: Front Plant Sci. 2024 Dec 23;15:1520786. doi: 10.3389/fpls.2024.1520786 (PMC11700743; doi:10.3389/fpls.2024.1520786)

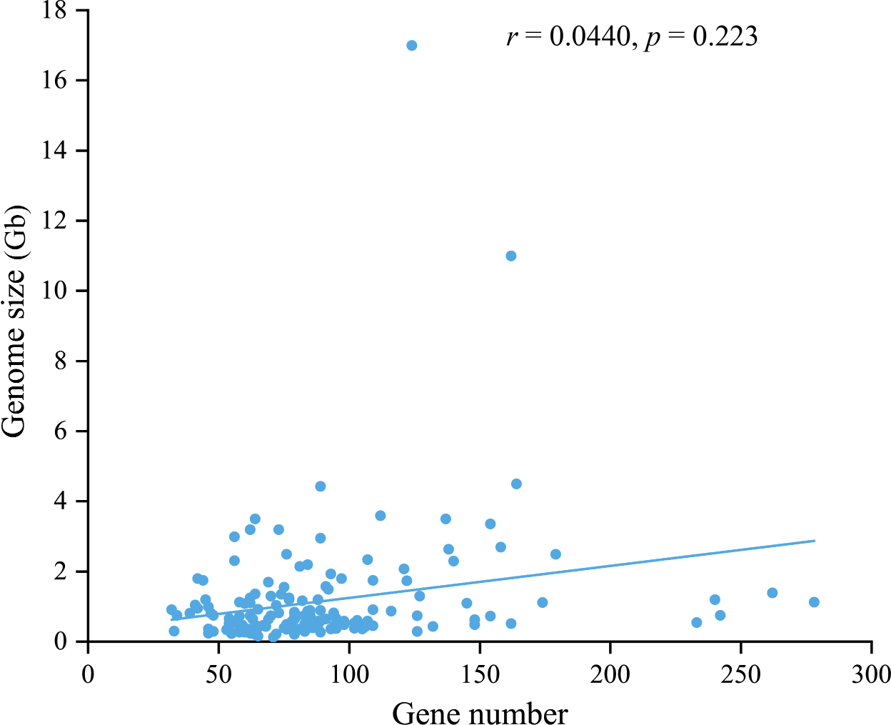

Supplement: Supplementary Figure 1 — Relationship between WRKY numbers in reported species and their corresponding genome sizes. [file Image1.tif]
